# 3D Collagen Hydrogel Promotes In Vitro Langerhans Islets Vascularization through ad-MVFs Angiogenic Activity

**DOI:** 10.3390/biomedicines9070739

**Published:** 2021-06-27

**Authors:** Monica Salamone, Salvatrice Rigogliuso, Aldo Nicosia, Simona Campora, Carmelo Marco Bruno, Giulio Ghersi

**Affiliations:** 1 Abiel s.r.l., c/o Department of Biological, Chemical and Pharmaceutical Sciences and Technologies (STEBICEF) University of Palermo, Viale delle Scienze, Ed. 16, 90128 Palermo, Italy; m.salamone@abielbiotech.com (M.S.); salvatrice.rigogliuso@unipa.it (S.R.); brunocarmelo@hotmail.it (C.M.B.); 2 Institute for Biomedical Research and Innovation-National Research Council (IRIB-CNR), Via Ugo La Malfa 153, 90146 Palermo, Italy; aldo.nicosia@irib.cnr.it; 3 Department of Biological, Chemical and Pharmaceutical Sciences and Technologies (STEBICEF) University of Palermo, Viale delle Scienze, Ed. 16, 90128 Palermo, Italy; simona.campora@unipa.it

**Keywords:** 3D coculture, angiogenesis, transplantation, islet of Langerhans, microvascular fragments

## Abstract

Adipose derived microvascular fragments (ad-MVFs) consist of effective vascularization units able to reassemble into efficient microvascular networks. Because of their content in stem cells and related angiogenic activity, ad-MVFs represent an interesting tool for applications in regenerative medicine. Here we show that gentle dissociation of rat adipose tissue provides a mixture of ad-MVFs with a length distribution ranging from 33–955 μm that are able to maintain their original morphology. The isolated units of ad-MVFs that resulted were able to activate transcriptional switching toward angiogenesis, forming tubes, branches, and entire capillary networks when cultured in 3D collagen type-I hydrogel. The proper involvement of metalloproteases (MMP2/MMP9) and serine proteases in basal lamina and extracellular matrix ECM degradation during the angiogenesis were concurrently assessed by the evaluation of alpha-smooth muscle actin (αSMA) expression. These results suggest that collagen type-I hydrogel provides an adequate 3D environment supporting the activation of the vascularization process. As a proof of concept, we exploited 3D collagen hydrogel for the setting of ad-MVF–islet of Langerhans coculture to improve the islets vascularization. Our results suggest potential employment of the proposed in vitro system for regenerative medicine applications, such as the improving of the islet of Langerhans engraftment before transplantation.

## 1. Introduction

Tissues are known to show a three-dimensional (3D) architecture that is difficult to recapitulate in bidimensional (2D) cell culture because it contains a well-organized vascular network, which usually results in a heterogeneous pattern distribution of metabolites, oxygen, and signalling molecules [1].

Natural biomaterials have been widely used to support the proliferation, adhesion, and migration of cells, and with this purpose, hydrogels are considered promising scaffolds due to their excellent swelling properties and their similitude to soft tissues [2]. Usually, they consist of a hydrophilic polymeric network of three-dimensionally cross-linked structures that absorb a substantial amount of water [3,4,5,6,7], in addition, three-dimensional (3D) printing is emerging as an innovative technique for generating 3D bioprinted collagen, gelatine, hyaluronic acid, or alginate-based hydrogel [8] The rapid technological advances made in this field appear to be promising for the development of hybrid hydrogels with enormous potential for application in the biomedical field and in regenerative medicine [9].

Systems allowing coculture of different cell types represent an attractive strategy to simulate the physiological environment, and it is not surprising that these systems been exploited in numerous basic and translational studies for studying the interactions between different cell types for industrial and medical applications [10]. Additionally, efforts have been made to reduce the use of animal models for large-scale screening and drug discovery. This motivated researchers to develop new 3D in vitro models to better simulate the in vivo microenvironment [11].

Based on biomaterial-inspired scaffolds, several studies addressed the possibility to repair tissue defects and injuries via tissue engineering application [12,13]. Moreover, the creation of preformed microvascular networks in the scaffolds before in vivo implantation which can improve engraftment efficiency represents a challenge [14,15]. Endothelial cells, endothelial progenitor cells and stem cells have been used for microvascular network construction [16,17]. Thus, it is possible to incorporate endothelial cells within a 3D scaffold allowing a rapid vascularization of the derivatives system [18,19]. In addition to stem cells, derivatives of adipose tissue, including microfat, nanofat, microvascular fragments (ad-MVFs), and stromal vascular fraction (SVF), may be used to improve the vascularisation of implants [20,21].

ad-MVFs are a mixture of arteriolar, capillary, and venous segments with variable lengths spanning from 20 to 150 μm [22,23]. They exhibit a whole microvascular morphology that includes the lumen, the endothelium, and perivascular cells containing endothelial cells, pericytes, and smooth muscle cells. Experimental studies revealed that ad-MVFs act as vascularization units for tissue engineering and regenerative medicine due to their angiogenic potential [22], which owes to the presence of proangiogenic cells and the release of the high amounts of angiogenic growth factors sustaining the vascularization [24]. Interestingly, ad-MVFs have shown the capability to rapidly reassemble in vivo, forming a functional microvascular network that can be exploited for several biomedical applications in cells therapy [25,26,27,28,29,30].

Among them, transplantation of islet of Langerhans represents a promising cell therapy for the treatment of type 1 diabetes to restore the canonical blood glucose homeostasis [31]. Preclinical models have shown that the restoration of blood flow to islets requires several days, maximizing the chance for ischemic conditions. This inadequate revascularization of transplanted islets is among the major causes of the reduction of islet function and engraftment [32].

As a proof of concept, we exploited ad-MVF–islet of Langerhans coculturing in 3D collagen hydrogel to improve the vascularization before transplantation and in turn to boost their engraftment. To optimize the in vitro cultivation of ad-MVFs before transplantation, we cultured them in 3D collagen type-I hydrogel. Here we show that, after a gentle dissociation of the adipose tissue, the culture of ad-MVFs in the type-I collagen hydrogel results in vessel morphology maintenance and angiogenesis promotion.

## 2. Materials and Methods

### 2.1. Isolation of ad-MVFs from Rat Adipose Tissue

Wistar female rats (average body weight of 350 g) were used for the vascular fragment extraction. The epididymal adipose tissue was minced in small pieces and mixed with a solution of Dulbecco’s Modified Eagle Medium (DMEM, Sigma, Milan, Italy) without serum, to which were added 150 U/gr Collagenase H (Abiel, Palermo, Italy) and 10 μg/mL Thermolysin (Promega, Madison, WI, USA) (10 mL/gr). Samples were incubated at 37 °C for 2 h in static condition.

After the incubation time, samples were centrifuged to remove adipocytes, and the pellet containing the vascular fraction was resuspended in 30 mL of DMEM with 10% (*v/v*) Foetal Bovine Serum (Euroclone, Milan, Italy) to stop the collagenase activity. The solution was then centrifuged at 300× *g*, 10 min at r.t. After discarding the supernatant, the pellet was resumed in 30 mL of DMEM 10% FBS and centrifuged at 300× *g*, 10 min at r.t. This step was repeated 3 times to wash the cells, and in the end, the pellet was resumed in 10 mL DMEM with 10% FBS.

### 2.2. ad-MVF Quantification

The amount of ad-MVFs obtained from a gram of treated adipose tissue was quantified. A dilution (1:10) of ad-MVF solution was seeded in a grid cell culture dish (NUNC^TM^, Roskilde, Denmark), and the number of vessels was evaluated by optical microscopy. The count was carried out 3 times for each sample of the 3 preparations (3 rats).

The ad-MVFs’ lengths were evaluated using ImageJ software (NIH Image, Bethesda, MD, USA) to obtain a range of different vessel sizes.

### 2.3. ad-MVF Culture Conditions, Viability, and Proliferation

The cell fraction containing vascular fragments was seeded both in 2D, on standard culture dishes, and in 3D, into the type-I collagen from rat tail gel (BD Biosciences, San Jose, CA, USA). The acid collagen solution (2.5 mg/mL) was prepared in Hank’s buffer (Sigma, Milan, Italy), and the pH was neutralized with 1N NaOH. ad-MVFs were resuspended into the neutralized solution, seeded on 24 well plates with a concentration of 1000 ad-MVFs/mL of collagen for well, and incubated at 37 °C, 5% CO_2_. After the pH neutralization, the collagen hydrogels were formed in few minutes. Complete DMEM medium was added to each sample.

According to the manufacturer recommendations, the viability and proliferation rates of ad-MVFs in 3D culture were quantified using the Alamar Blue colourimetric assay (Thermo Scientific, Waltham, MA, USA). The amount of developed fluorescence was evaluated (excitation/emission wavelengths of 530/590 nm) using the plate reader DU-730 Life Science spectrophotometer. The analysis was carried out in triplicate for each preparation (n 3 rat).

### 2.4. Isolation of Pancreatic Islets

The pancreatic islets were obtained from processing the pancreases harvested from 350 g Wistar female rats using a classical surgical procedure [33]. An enzyme blend consisting of recombinant collagenase G (40 units) and collagenase H (170 units) (Abiel, Palermo, Italy) plus 100 micrograms of thermolysin (Promega, Milan, Italy) in 10 mL was used [34,35,36,37,38,39].

The enzyme blend was prepared immediately before the perfusion in the pancreatic duct using a needle-cannula. After 5 mL enzyme blend perfusion, the pancreas was harvested, transferred into a tube containing the other 5 mL of digestion solution, and incubated for 10 min at 37 °C in a water bath. At the end of the incubation time, the sample was moved to ice, and 10 mL of cold RPMI1640 (Sigma, Milan, Italy) with 10% (*v/v*) FBS was added to stop the enzyme digestion.

The pancreatic tissue was completely dissociated by vigorous shaking of the tube (40 times in 10 s). This step was critical for optimal islet recovery and lowering the number of exocrine cell clusters. Therefore, the tube was centrifuged for 2 min at 800 rpm at 4 °C; the supernatant was carefully poured off without disturbing the pellets, which were resuspended in 20 mL of cold medium with 10% (*v/v*) FBS.

The sample was poured off through a 380 μM (S0770, Sigma, Milan, Italy) wire mesh into a fresh Petri dish. The initial tube was rinsed with an additional 10 mL of media and poured through the wire mesh over the nondigested tissue. The filtrate was transferred to a fresh 50 mL tube. Digested tissue was dissolved in 15 mL ice-cold heavy Histopaque (Sigma, Milan, Italy) (1.119 g/mL) and transferred to a new 50 mL tube. To ensure that the suspension was homogeneous, an additional 20 mL layer of ice-cold light Histopaque (1.077 g/mL) was carefully added. Histopaque was carefully overlayed with 15 mL of ice-cold serum-free RPMI 1640. After 5 min of 800× *g* centrifugation at 4 °C using slow acceleration and prolonged breaking time, the islets migrated to the interface between the serum-free media and Histopaque (1077 g/mL), while the exocrine cells formed a pellet at the bottom of the tube.

Purified islets were collected in a 50 mL centrifuge tube prefilled with 15 mL of cold RPMI and filled up with cold RPMI to 50 mL. The tube was centrifuged for 2 min at 800 rpm at 4 °C. The supernatant was poured off, and the islets were resuspended in 10 mL of RPMI 1640 containing 10% (*v/v*) FBS, to which was added 100 units/mL penicillin G and 100 µg/mL streptomycin (Euroclone), and put in a 10 cm petri dish at 37 °C in a humidified atmosphere of 5% CO_2_ and maintained in sterile conditions.

### 2.5. Coculture of Islets and ad-MFVs in Collagen Type-I Hydrogel

Islet and ad-MVFs were cocultured within 3D rat collagen type-I hydrogel (BD Biosciences, San Jose, CA, USA). Collagen solution was prepared in Hank’s buffer (2.5 mg/mL) and neutralized with NaOH to allow collagen polymerization. Immediately after, 10,000 ad-MVFs were resuspended into 10 mL of neutralized collagen solution containing 300 islets. One millilitre of hydrogel was seeded for each well of 24 well plates and incubated at 37 °C with 5% CO_2_. After polymerization, 1 mL of complete RPMI (Sigma, Milan, Italy) was added.

### 2.6. Glucose Stimulated Insulin Secretion (GSIS) Assay

The functionality of islets in 3D collagen hydrogel was evaluated by measuring insulin release upon glucose stimulation. To this aim, the medium from the hydrogel was removed, and 1 mL of 3.3 mM glucose in Krebs buffer solution was added. The medium was changed 3 times every 10 min.

One millilitre of Krebs buffer containing 3.3 mM glucose was added, and the mixture was incubated for 60 min. The medium was removed and stored at −20 °C for further usage. A second incubation was performed with the Krebs solution containing 16.7 mM glucose for 60 min. Finally, the medium was stored at −20 °C.

Insulin quantification was performed by using the insulin quantification assay (Mercodia, Uppsala, Sweden). The GSIS index represents the ratio of insulin secreted at 16.7 mM glucose and the insulin secreted at 3.3 mM glucose.

### 2.7. Western Blots

Sodium dodecyl sulphate–polyacrylamide gel electrophoresis (SDS–PAGE) was carried out as shown by Laemmli. Lysates obtained from ad-MVFs on collagen hydrogels (20 μg/sample) were subjected to SDS–PAGE under reducing conditions and finally transferred on a nitrocellulose membrane through electroblotting at 100 V and 300 mA for 90 min at low temperature (Hybond, Amersham). After protein transfer, the membrane was saturated with 3% nonfat dry milk in TBS-T (0.6% Trizma base; 0.87% NaCl; 0.05% Tween 20).

The target proteins were recognized by the following monoclonal antibodies in TBS-T: Rabbit anti-CD31 (ab28364); Mouse anti-CD90 (ab225); Mouse anti-αSMA (F3777, Sigma). Mouse mAb against β-actin (0.5 μg/mL; A2228, Sigma) was used to normalize protein content. HRP-conjugated antimouse and antirat (Sigma) were diluted in TBS-T (1:25,000 and 1:80,000, respectively) and adopted as the secondary antibody. The membrane was exposed to Super Signal West Femto Maximum Sensitivity substrate (Thermo Scientific), and protein expression was detected by ChemiDoc XRS (Biorad, Hercules, CA, USA).

### 2.8. Zymography

Zymography was performed on substrate gelatine supplemented–PAGE. The hydrogel containing cells at a different time was extracted in RIPA buffer (Sigma) using manual homogenization with a Teflon pestle, and protein quantification was performed after extraction using Bradford colourimetric assay. Lysates obtained from ad-MVFs on collagen hydrogels (20 μg/sample) at a different time were loaded on SDS–PAGE under native conditions. After electrophoresis, gelatine zymographies were incubated for 24 h at 37 °C in two developing buffers: activator buffer containing 2 mmol/L CaCl2, Tris-HCl buffer (50 mmol/L; pH 7.4) containing 1.5% Triton X-100 and 0.02% Na Azide, to activate gelatinases; and inhibitor buffer Tris-HCl buffer (50 mmol/L; pH 7.4), containing 1.5% Triton X-100 and 0.02% Na Azide plus 2 mmol/L EDTA, to inhibit gelatinase activities. After incubation, gels were stained using Coomassie Brilliant Blue G-250 (Sigma).

### 2.9. Immunofluorescence Assay

ad-MVFs and Langerhans islets in 2D conditions and 3D collagen hydrogels containing in coculture ad-MVFs and islets were fixed in 3.7% formaldehyde (Sigma) for 10 min and permeabilized with 0.1% Triton X-100 (Sigma) for 5 min. The target proteins were recognized by the following monoclonal primary antibodies: Rabbit CD31 (1:500) (Abcam, Cambridge, UK), Mouse CD90 (1:300) (Abcam), and Mouse αSMA FITC conjugated (Sigma). In the same cases, αActin was detected using FITC-labelled phalloidin (1:300) (Sigma). The cell nuclei were stained by DAPI (1:20.000) (Sigma). Cells in 2D and 3D conditions were respectively observed under an OLYMPUS BX50 Fluorescence microscope and OLYMPUS IX70 confocal microscope.

### 2.10. RNA Isolation and cDNA Synthesis

Total RNA was purified using Trizol (Invitrogen, Waltham, MA, USA) according to the manufacturer’s instructions from newly isolated ad-MVFs and ad-MVFs cultured on 3D collagen type-I hydrogel at day 1 and day 8.

RNA concentrations and quality were verified by measuring the optical density at Abs260 nm and Abs260/280 nm. The RNA integrity was evaluated on denaturing 1.5% agarose gel. To remove DNA contamination, 500 ng of extracted RNA was treated with DNase RQ1 RNase-Free (Promega, Madison, WI, USA) for 30 min at 37 °C. DNase was then inactivated by adding 25 mM EDTA.

First-strand cDNA was synthesized from 250 ng DNase-treated RNA using a High-Capacity cDNA Reverse Transcription Kit (Life Technologies Corporation, Carlsbad, CA, USA) according to the manufacturer’s instructions. The cDNA mixtures were stored at −20 °C.

### 2.11. RT-qPCR Analyses

RT-qPCR was performed using the BIO-RAD CFX96 System with BrightGreen 5X qPCR MasterMix (Applied Biological Materials Inc, Richmond, BC, Canada) as detection chemistry. Real-time PCRs were carried out in a 20 µL mixture containing 1 µL of a 1:10 dilution of the cDNA preparations. The following parameters were used for qPCR: 95 °C for 10 min, followed by 40 cycles of 95 °C for 10 s and 60 °C for 60 s, followed by melting curve analysis and electrophoresis on 2% agarose gels to confirm the absence of nonspecific products.

Primer pairs are shown in Table 1. *Gapdh* was chosen as a reference gene and used to quantify target genes expression levels using the ΔΔCt method. Amplifications were run in triplicate. All data represented relative mRNA expressed as the mean ± S.D. (*n* = 3). Significant differences between the treated groups and the reference control were determined by a *t*-test using Statistica 6.0 (StatSoft, Tulsa, OK, USA).

## 3. Results

### 3.1. ad-MVF Isolation and Culture in 3D Collagen Type-I Hydrogel

**Figure 1:**
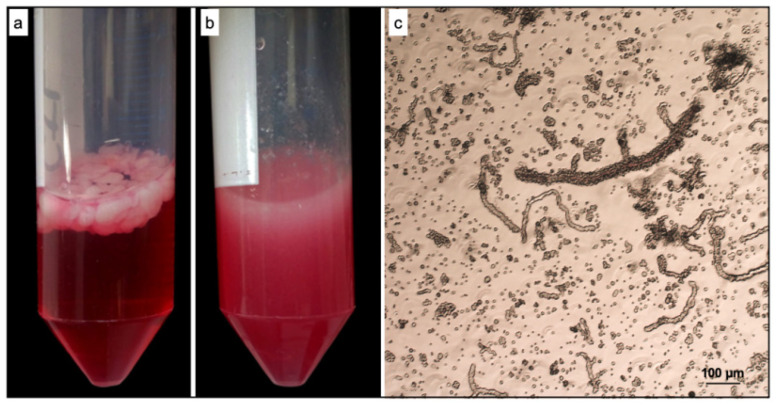
Isolation of stromal vascular fragments (ad-MVFs) from rat adipose tissue. (**a**) Rat adipose tissue before digestion. (**b**) Rat adipose tissue after digestion. (**c**) Optical microscopy image of freshly isolated ad-MVFs.

Gentle tissue dissociation is a critical procedure due to the difficulty of maintaining the cell surface epitopes. As reported in the experimental section, a gentle and efficient tissue dissociation method was developed to generate stromal vascular fraction from rat adipose tissue containing microvascular fragments (ad-MVFs) (Figure 1).

**Figure 2:**
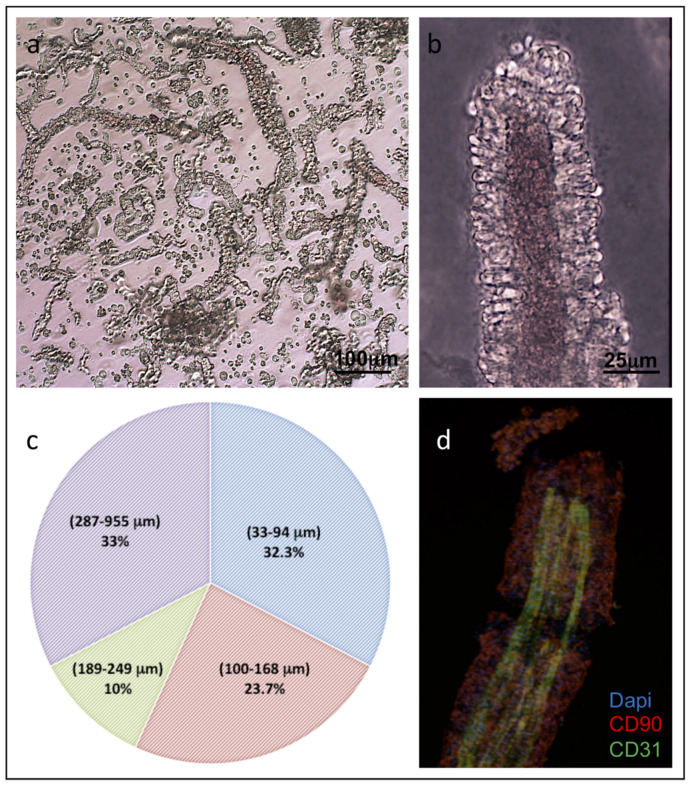
Characterisation of isolated ad-MVFs. (**a**) Optical microscopy of the ad-MVFs; (**b**) optical microscopy of ad-MVF showing larger calibre. (**c**) The percentage distribution of isolated ad-MVFs with lengths ranging from 32.3–955 μm. (**d**) Immunohistochemical localisation of CD31 (green) in the endothelial cells of the vascular lumen and CD90 (Red) in the pericytes of the perivascular wall. Cell nuclei were stained with DAPI (blue).

The isolated ad-MVFs were quantified as described in the Material and Methods section. From each preparation, it was possible to obtain about 1500 ad-MVFs/gr consisting of a mixture of vessels with a length distribution between 32.3 and 955 μm. (Figure 2a–c). The immunohistochemical (IHC) evaluation of ad-MVFs to detect CD31, a marker of the lumen endothelial cells, and CD90, a marker of perivascular stabilizing cells such as pericytes, demonstrated the maintenance of an intact microvascular morphology (Figure 2d).

To evaluate their viability, the isolated ad-MVFs were first seeded on plates and cultured in a 2D system for up to 8 days: in 2D, they started to proliferate soon with a consequent spread of cells on the substrate, resulting in the loss of the vessel morphology (data not shown).

**Figure 3:**
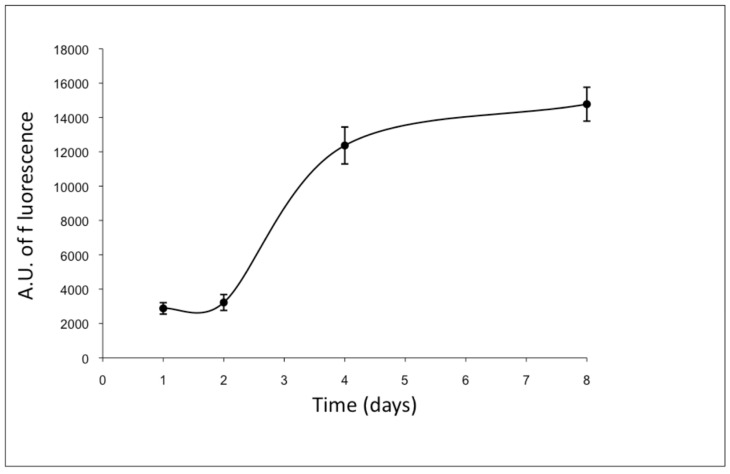
Proliferation curve of ad-MVF embedded in type-I collagen 3D hydrogel (Almar blue assay). Cells began to actively proliferate from the second day of culture and reached the capillary network after 8 days. The fluorescence values were expressed as arbitrary units (A.U.).

To maintain their original morphology, ad-MVFs were cultured on collagen type-I hydrogel: collagen fibrils, if placed at 37 °C in neutral pH conditions, show the ability to self-assemble, forming a stable hydrogel. In this manner, the cells of ad-MVFs began to actively proliferate and reached a complex capillary network after 8 days of culture (Figure 3).

**Figure 4:**
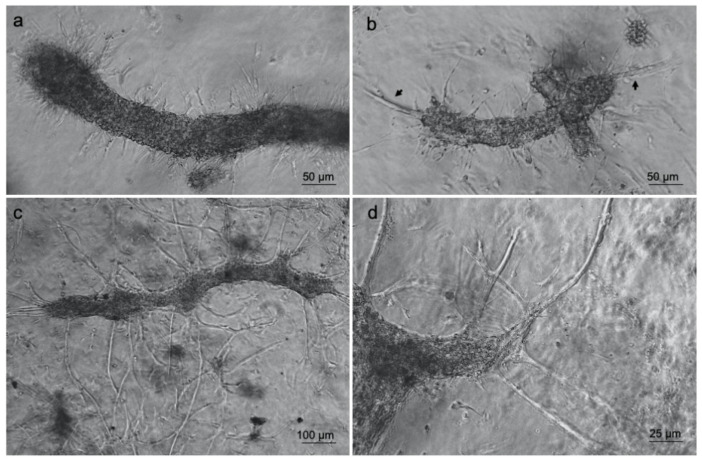
Type-I collagen 3D hydrogel supports ad-MVF sprouting. Optical microscopy images of ad-MVFs cultured in 3D collagen hydrogel at 2 days (**a**,**b**) and 8 days (**c**,**d**). Arrows in (**b**) indicate sprouting from tip cells.

Furthermore, optical microscopy analyses revealed that some cells started to change their original morphology, encompassing an endothelial to mesenchymal transition by producing membrane protrusions such as filopodia (Figure 4a) by forming new sprouts from tip cells (Figure 4b) and thus supporting the generation of new arms of the vessels (Figure 4c,d). The generation of membrane protrusions occurred either along or at the vessels’ opposite ends. Once the process was finished, the formation of a new capillary network was obtained as shown in Figure 4c.

To assess the suitability of type-I collagen hydrogel in providing an adequate substrate for angiogenesis, the proper involvement of enzymes belonging to the metalloprotease and serine protease family in the degradation of the basal lamina during the angiogenesis process was evaluated. Therefore, zymography analyses were carried out to analyse the activation/expression of gelatinases produced by the ad-MVFs after 3D culturing at various time points corresponding to day 1 (endothelial cells start to change morphology and degrade the basal lamina), day 4 (cells invade the collagen hydrogel and new arms were generated), and day 8 (new capillary network is established).

**Figure 5:**
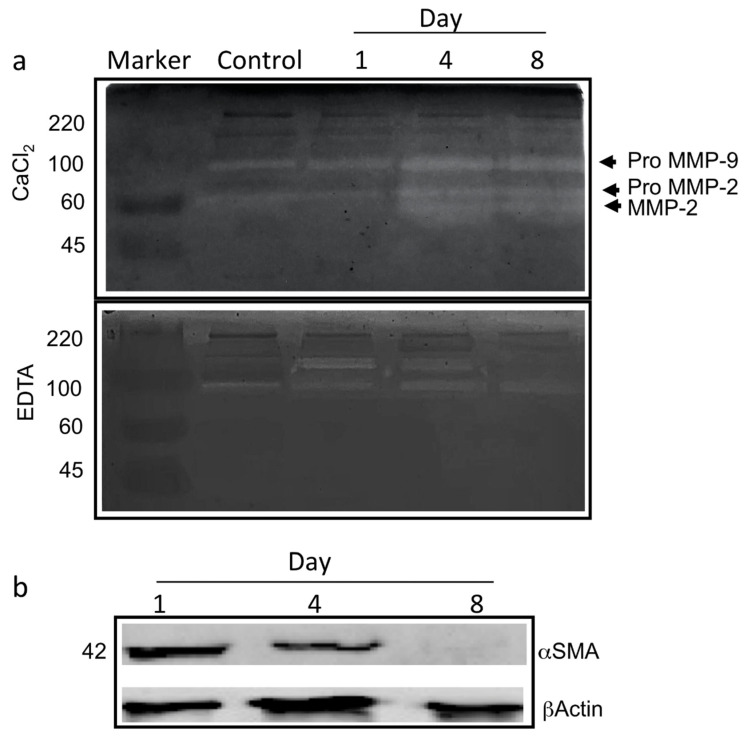
The angiogenesis in ad-MVFs occurred via the involvement of gelatinases and αSMA reduction. (**a**) Gelatine zymography (with CaCl_2_ or EDTA) of proteins extracted from ad-MVFs cultured in 3D collagen hydrogel after 1 day, 4 days, and 8 days; protein extracted from 3D collagen hydrogel without cells was used as a negative control (Control). Activation of proMMP9 and proMMP2 is shown. (**b**) Western blotting analysis of proteins extracted from ad-MVFs cultured in 3D collagen hydrogel after 1, 4, and 8 days. A reduction in the total amount of αSMA is shown.

The gelatinolytic pattern is reported in Figure 5a. On day 4 and day 8, the occurrence of digestion bands of increased intensities ranging from 60–100 KDa, corresponding to pro-metalloprotease-2 (proMMP2) and pro-metalloprotease-9 (proMMP9), respectively, was evident. Additionally, it appeared that the activated form of MMP-2 was generated starting from day 4.

Inhibition tests in the presence of EDTA (Figure 5a) showed that digestion bands corresponding to MMP2 and proMMP9 largely disappeared. In contrast, a calcium-independent band of about 170 KDa likely corresponding to Seprase was measured on day 1, simultaneously with the observed changes in cell morphology and the basal lamina degradation. Interestingly, the signal decreased after 4 days of 3D culturing, while the proteolytic activity nearly disappeared on day 8.

To confirm that the hydrogel herein proposed provides an adequate 3D environment supporting the activation of angiogenic programs, the expression of alpha-smooth muscle actin (αSMA) was evaluated by Western blot analysis. ad-MVFs expressed higher αSMA levels on day 1, while a significant reduction in the protein amount was measured on both day 4 and day 8 (Figure 5b).

### 3.2. Gene Expression Analyses of ad-MVFs in 3D Collagen Type-I Hydrogel

**Figure 6:**
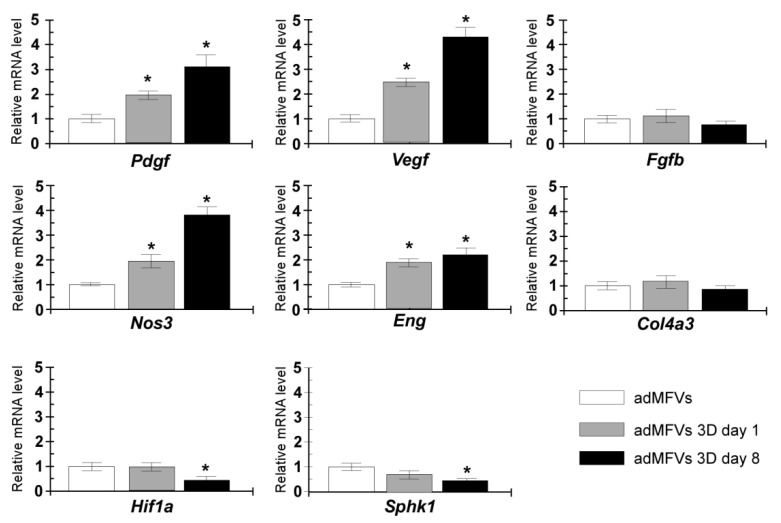
Gene expression analyses of *Col4a3*, *Nos3*, *Pdgf*, *Fgfb*, *Vegf*, *Sphk1*, *Hif1a*, and *Eng*. The RT-qPCR assays were performed in newly extracted ad-MFVs and sprouting ad-MFVs at day 1 and day 8 in 3D collagen type-I hydrogel. The gene expression levels were analysed using the 2-ΔΔCt method using Gapdh as the reference gene. The data represent the mean ± SD of three independent culture experiments. Bars with an asterisk are significantly different at *p* < 0.05.

To further confirm the angiogenic regulatory switching toward sprouting, the transcriptional profile of eight genes recognized to exert a functional role in angiogenesis was analysed. The transcriptional levels of collagen type-IV alpha 3 chain (*Col4a3*), nitric oxide synthase 3 (*Nos3*), platelet-derived growth factor (*Pdgf*), basic fibroblast growth factor (*Fgfb*), vascular endothelial growth factor (*Vegf*), sphingosine kinase 1 (*Sphk1*), hypoxia-inducible factor 1-alpha (*Hif-1a*), and *endoglin* (*Eng*) were evaluated in ad-MVFs cultured on 3D collagen hydrogel at days 1 and 8 and compared with those observed from newly extracted ad-MVFs (Figure 6).

The ad-MVFs cultured on 3D collagen hydrogel at day 1 showed upregulation of several genes, including *Pdgf*, *Vegf*, *Nos3*, and *Eng*, while the transcript levels of *Fgfb*, *Col4a3*, *Hif-1a*, and *Sphk1* remained unchanged. Sprouting ad-MVFs (day 8) showed a significant increase in the mRNA levels of *Nos3*, *Pdgf*, *Vegf*, and *Eng* (3.8-fold, 3.1-fold, 4.9-fold, and 2.1-fold, respectively), while transcript levels of *Col4a3* and *Fgfb* remained similar to those observed in newly extracted ad-MVFs. Conversely, the RT-qPCR analysis measured a significant reduction in the *Sphk1* and *Hif-1a* transcript levels after 8 days for 3D cultured ad-MFVs.

### 3.3. Islet of Langerhans and Ad-SVF Coculture into Type-I Collagen Hydrogel

**Figure 7:**
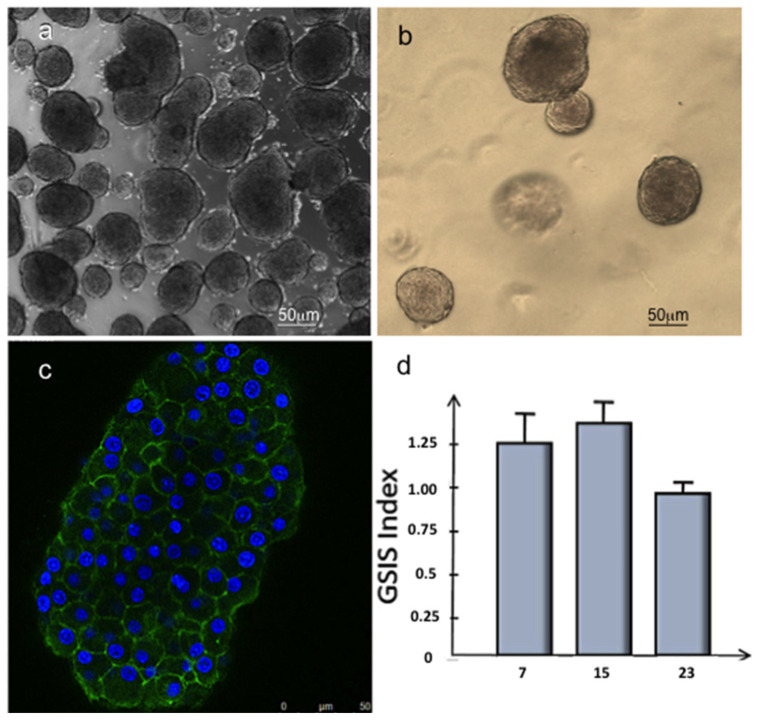
Morphological and metabolical analysis of Langerhans islets. Optical microscopy images of Langerhans islet cultured respectively in 2D conditions after 24 h (**a**) and in 3D collagen hydrogel (**b**) after 15 days. (**c**) Immunofluorescence analysis of Langerhans islet stained with phalloidin-FITC (green) and DAPI (blue) after 15 days of culture in 3D collagen hydrogel. (**d**) GSIS index (the ratio of insulin secreted at 16.7 mM glucose and the insulin secreted at 3.3 mM) of Langerhans islets cultured in 3D collagen hydrogel after 7, 15, and 23 days.

Islets of Langerhans have been shown to gradually disintegrate after culturing in 2D conditions in a time-dependent manner; this is related to the floating condition of the islets [40]. For this reason, to develop a biocompatible scaffold supporting islet of Langerhans vascularisation, 3D collagen hydrogel was preliminarily tested for the maintenance of viable islets in culture. The islet phenotype was observed after 24 h (Figure 7a) and 15 days of 3D culture within type-I collagen hydrogels (Figure 7b).

The morphological analyses carried out on the islets of Langerhans showed that the islets kept the basal lamina surrounding them, which appeared well intact even after the enzymatic digestion process of the pancreas rat with the canonical architecture with well-preserved cell–cell interactions (Figure 7b,c).

To further confirm such healthy status maintenance, the islets were cultured in 3D collagen hydrogel for 3 weeks. They were functionally tested to release insulin in response to glucose stimulus (Figure 7d).

The 3D cultured islets showed GSIS indices (the ratio of insulin secreted at 16.7 mM glucose and the insulin secreted at 3.3 mM) comparable to those of functional islets (superior to 1), thus confirming that the mechanism of secretion was well preserved at least until day 15 (Figure 7d).

**Figure 8:**
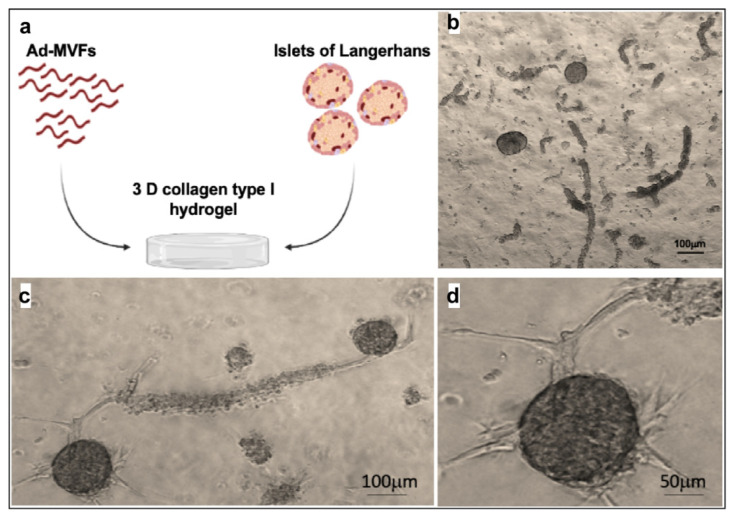
Langerhans islets and ad-MVFs cocultured in 3D collagen hydrogel. (**a**) Schematic representation of the coculture system. (**b**) Optical microscopy image of islets and ad-MVFs cocultured in 3D collagen hydrogel at day 0. (**c**) Optical microscopy image of Langerhans islets and ad-MVFs cocultured in 3D collagen hydrogel at day 8. (**d**) Magnification of a vascularized islet at day 8.

Once Langerhans islet biocompatibility was established on this hydrogel, the ad-MVFs’ capability to vascularize the islets of Langerhans in 3D collagen hydrogel was evaluated. Thirty IEQ (islets equivalent) were mixed with 1000 ad-MVFs and embedded in 1 mL of the collagen hydrogel (Figure 8a,b). Endothelial tip cells started to grow from existing ad-MVFs and guided the developing capillary sprout, which was finally connected to the islets after 8 days in culture (Figure 8c,d).

**Figure 9:**
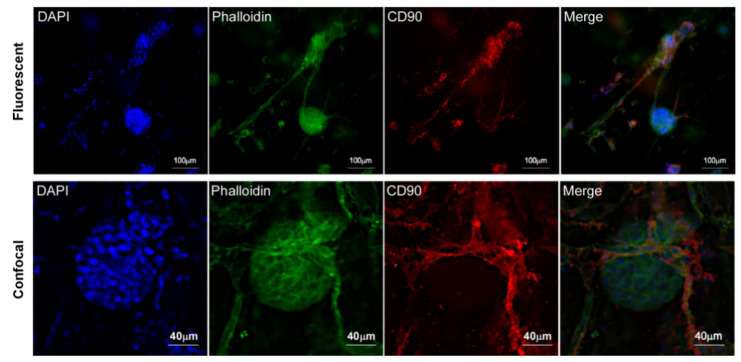
Fluorescence and confocal microscopy images of islet vascularized by ad-MVFs after coculturing in 3D collagen hydrogel at day 8. CD90 expression is localized on ad-MVF cells’ surface. Blue: DAPI; green: phalloidin-FITC; red: CD90.

To confirm the vascularisation, fluorescence IHC staining of CD90 was carried out in ad-MVF and islets of Langerhans 3D coculture. As shown in Figure 9, CD90 localized only at the membrane level of new vessels and pericytes. Instead, it was possible to observe the localization of actin in the cortical area of both vessels and islets. Furthermore, confocal microscopy analysis strongly suggested that CD90 was expressed by activated endothelial cells and pericyte membrane protrusions surrounding the islet (Figure 9).

## 4. Discussion

ad-MVFs are usually isolated from adipose tissue via tissue digestion in the presence of a blend of enzymes containing an undefined ratio of collagenase G and H from *Clostridium histolyticum* in addition to several residual protease activities [41].

Tissue digestion represents a critical step in several isolation protocols, including that for ad-MVFs, because the isolated component may lose the original morphology and function if the enzyme blend used is too aggressive. This is probably due to the adipose tissue dissociation that is usually carried out using an enzyme blend containing undefined activity of proteases; thus, in order to preserve the morphology and the activity of the cells, only brief treatments (10–15 min) are allowed.

To improve either yield or quality of extracted ad-MVFs, an isolation procedure was optimized. We herein show for the first time that highly pure recombinant collagenase H can digest native adipose tissue as obtained for viable chondrocyte isolation from cartilaginous tissues [42]. Moreover, it should be noted that the ad-MVFs isolated with this procedure showed higher median length and maintained intact microvascular morphology, including a lumen, an endothelium, and a perivascular structure (33 to 955 μm vs. 20 to 150 μm) [41].

The in vitro ad-MVF culture is also a critical step for the maintenance of the functional 3D structure. For this reason, ad-MVFs are generally transplanted immediately after isolation [29].

McDaniel et al. showed that cells derived from ad-MVF explants exhibited higher mesenchymal stem cells phenotypes and capacity for angiogenic differentiation rather than ASCs [24]. For this reason, ad-MVFs may produce beneficial effects that extend beyond their capability to support blood flow in vivo. Three-dimensional cultures and organoid studies are used in basic research to study cell biology and physiology under more realistic conditions [43].

ad-MVFs are generally transplanted immediately after isolation since they can easily lose their original morphology when cultivated in a 2D system. Thus, efforts were made to overcome such limitations. Herein we show that ad-MVFs cultured in 3D collagen hydrogel can support new angiogenesis without losing the vessel morphology. A vascular network expansion was developed from existing ad-MVFs mainly by sprouting new branches that connect and subsequently remodel into a functional vascular circuit. At the cellular level, a beginning sprouting angiogenesis was individuated that permits to cells to break out of the vessel wall, degrade the basement membrane, proliferate, and together invade the surrounding tissue. The tip cells degrade the extracellular matrix by releasing matrix metalloproteinases (MMPs) and migrate into the surrounding tissue [44,45]. MMPs, inhibitors of MMPs, and serine endopeptidases, including seprase and DPPIV, are known to be involved in remodelling of the ECM during angiogenesis [46,47,48,49]. MMPs also contribute to angiogenesis by removing pericytes from vessels, releasing angiogenic growth factors from ECM, and confining on the cell surface of the invading tips of migrating endothelial cells by generating functional ECM fragments and by cleaving VE-cadherin to break endothelial cell–cell adhesions [47]. MMPs and serine endopeptidase are also involved in the angiogenesis process by their localization on cellular protrusion and membrane vesicles [50,51,52].

In the proposed system, during the different steps of angiogenesis, changes were observed in the expression and activation of both metalloproteases MMP2 and MMP9 and a gelatinase calcium-independent presumably corresponding to seprase. Furthermore, a decrease of αSMA levels was observed, probably because the new small vessels were at the earlier stage of the newly developed microvascular networks and were composed mainly of endothelial cells [53,54]. Moreover, αSMA, normally associated with large vessels, has been reported to decrease during the angiogenetic process [55].

It is recognized that the angiogenic “switch on” mainly relies on the upregulation of proangiogenic factors and/or downregulation of angiogenesis inhibitors [45,56,57]. Among factors positively involved in angiogenesis, the *VEGF*, *FGF*, and *PDGF* family members mediate their angiogenic effects by binding to specific tyrosine kinase receptors and triggering several downstream signalling pathways that in turn regulate the expression of selected genes contributing to sprouting [58]. Transcriptional upregulation of *VEFG* and *PDGF* has been shown in various in vitro and in vivo angiogenesis models [59,60,61].

ad-MFVs cultured in 3D type-I hydrogel expressed increased mRNA levels of *VEGF* and *PDGF* compared to controls, while the expression of *Col4a3*, which has been referred to as a suppressor of angiogenesis via tumstatin [62], remained unchanged. These results suggest the activation of a proangiogenic signalling network that is likely responsible for the induced sprouting. In this line, it has been shown that *eNOS* plays a pivotal role in mediating *VEGF*-related angiogenesis and endothelial function via the production of nitric oxide (NO) [63,64]. Moreover, *eNOS* was upregulated in response to *VEGF-A*. Thus, it is not surprising that 3D cultured ad-MVFs also expressed increased *Nos3* (previously named *eNOS*) mRNA levels likely related to *VEGF* signalling.

Additionally, it should be noted that bFGF-induced angiogenesis did not rely on NO. In the experimental settings, no changes occurred in the *bFGF* transcript levels in sprouting microvessel, whereas *Eng*, which is involved in *VEGF*-induced angiogenesis [65], was upregulated. Therefore, it is reasonable to hypothesize that collagen type-I hydrogel provided an adequate 3D environment supporting the activation of a *VEGF*-mediated angiogenesis in ad-MVFs.

Moreover, it was noteworthy that *Sphk1* and *Hif1a* were downregulated in ad-MFVs after culturing in a 3D collagen scaffold. Although both factors are known to be positively involved in the activation of angiogenesis, it has been proposed that upregulation of *Sphk1*, which acts upstream of *Hif1a*, occurs under hypoxic conditions [66,67]. Hence, evidence supporting proper oxygen diffusion through the collagen scaffold also emerged.

To the best of our knowledge, only two studies showed the use of ad-MVFs to improve islet transplantation [30,68]. However, no biochemical or molecular characterizations were provided by Hiscox and colleagues [30], while Nalbac immediately transplanted the hybrid system in absence of a collagen matrix [68]. Other clinical trials have shown that the coculture of islets of Langerhans with stem cells preserves the tissue integrity and functionality of the tissue construct [69]. The development of new blood vessels originating from single endothelial cells requires up to 10 days [14], which is not fast enough to guarantee islets’ survival in the initial phase after transplantation.

The angiogenetic potential of the system was exploited to vascularize the islet of Langerhans. The suitability of collagen type-I hydrogel in the maintenance of islet viability and function was herein shown by embedding islets in 3D collagen hydrogel and analysing their capability to produce insulin under glucose stimuli.

Moreover, when seeded with ad-MVFs in the collagen hydrogel, the islets became progressively vascularized by new vessels. This is important because β-cells directly interact with the capillaries, suggesting that increased vascularization help β-cells to rapidly respond to the blood glucose increase by secreting insulin, but also stimulate β-cell proliferation [70]. This spatiotemporal process was facilitated by the heterogeneous spatial distribution pattern of oxygen, metabolites, and signalling molecules in a 3D type-I collagen hydrogel system, making the system more similar to the in vivo pancreas tissue.

## 5. Conclusions

ad-MVFs obtained via a gentle dissociation from adipose tissue being cultured in type-I collagen hydrogel resulted in the maintenance of vessel morphology as well as in the promotion of the angiogenic process. This occurred via the activation of MMP2 and MMP9 and reduction of αSMA likely related to basal lamina dismantling and the generation of a new small immature vascular network. Additionally, gene expression analyses suggested the activation of *VEGF*-related angiogenesis. The angiogenetic potential of this system was exploited in the vascularization of the islet of Langerhans. Once Langerhans islet viability and functionality when growing on type-I collagen hydrogel was established, the capability of ad-MVFs to vascularize the islets was verified; the obtained results suggested translational applications in the field of regenerative medicine and tissue engineering. In addition, 3D coculturing represents an efficient in vitro system resembling a physiological scenario that can be used to evaluate structural and functional interactions among different cell types and tissues. We believe that this system can be used for the study of the crosstalk among islet and endothelial cells in response to microenvironment changes including physiological glucose homeostasis [71].

## Figures and Tables

**Table 1:** Oligonucleotide primers used in this study.

Genes	Sequences (5′–3′)	Accession Number
*Gapdh*	CAGCCTCGTCTCATAGACAAGATG ^a^ AAGGCAGCCCTGGTAACCA ^b^	AF106860
*bFgf*	GAGAGAGGAGTTGTGTCCATCAAG ^a^ GCAGCCGTCCATCTTCCTT ^b^	X61697
*Pdgfb*	TGGAGTCGAGTCGGAAAGCT ^a^ GAAGTTGGCATTGGTGCGAT ^b^	NM_031524.1
*Vegfb*	GAGGAAAGGGAAAGGGTCAAAA ^a^ CACAGTGAACGCTCCAGGATT ^b^	AF062644
*Hif1a*	GTTTACTAAAGGACAAGTCACC ^a^ TTCTGTTTGTTGAAGGGAG ^b^	NM024359
*Nos3*	GACCCTCACCGATACAACATAC ^a^ CATACAGGATAGTCGCCTTCAC ^b^	NM_021838
*SphK1*	TCAGTCTGTCCTGGGGTTTC ^a^ TCCTCCAGAGGAACGAGGTA ^b^	NM_001270811.1
*Col4a3*	CCCTTGAGCCCTACGTTAGCA ^a^ CCTCAGAGCCTGCACTTGTAAACA ^b^	XM_343607
*Eng*	TGCTCCCTCTGGTCATTACC ^a^ CCTGGCTGGTGGTGTATGTC ^b^	NM_001010968

^a^ Forward primer, ^b^ Reverse primer.

## Data Availability

Not applicable.
